# Family-Based Association Test Using Both Common and Rare Variants and Accounting for Directions of Effects for Sequencing Data

**DOI:** 10.1371/journal.pone.0107800

**Published:** 2014-09-22

**Authors:** Ren-Hua Chung, Wei-Yun Tsai, Eden R. Martin

**Affiliations:** 1 Division of Biostatistics and Bioinformatics, Institute of Population Health Sciences, National Health Research Institutes, Zhunan, Miaoli, Taiwan; 2 Hussman Institute for Human Genomics, University of Miami Miller School of Medicine, Miami, Florida, United States of America; University of North Carolina, United States of America

## Abstract

Current family-based association tests for sequencing data were mainly developed for identifying rare variants associated with a complex disease. As the disease can be influenced by the joint effects of common and rare variants, common variants with modest effects may not be identified by the methods focusing on rare variants. Moreover, variants can have risk, neutral, or protective effects. Association tests that can effectively select groups of common and rare variants that are likely to be causal and consider the directions of effects have become important. We developed the Ordered Subset - Variable Threshold - Pedigree Disequilibrium Test (OVPDT), a combination of three algorithms, for association analysis in family sequencing data. The ordered subset algorithm is used to select a subset of common variants based on their relative risks, calculated using only parental mating types. The variable threshold algorithm is used to search for an optimal allele frequency threshold such that rare variants below the threshold are more likely to be causal. The PDT statistics from both rare and common variants selected by the two algorithms are combined as the OVPDT statistic. A permutation procedure is used in OVPDT to calculate the p-value. We used simulations to demonstrate that OVPDT has the correct type I error rates under different scenarios and compared the power of OVPDT with two other family-based association tests. The results suggested that OVPDT can have more power than the other tests if both common and rare variants have effects on the disease in a region.

## Introduction

Genome-wide association studies (GWAS) have been successful in identifying common variants associated with complex diseases. However, most identified variants explain only a small portion of heritability for the complex trait [1]. The missing heritability may be explained by rare variants, which can now be efficiently generated by studies using the next-generation sequencing (NGS) technology, such as the 1000 Genomes Project [2]. Moreover, with the advancements in NGS, sequencing a large number of individuals for an association study has become possible. Thus, the development of statistical association tests for the analysis of rare variants has become important.

Association tests for rare variants have been developed rapidly for case-control studies. The cohort allelic sums test (CAST) was the first test developed specifically for rare variant association analysis [3]. For the test, counts of mutant alleles were summed over a region (e.g., a gene or an exon), and the difference in allele frequencies for the collapsed alleles between cases and controls is tested. The combined multivariate and collapsing (CMC) test extended the CAST to jointly test both common variants and groups of rare variants [4]. The weighted-sum test further extended the CAST to assign informative weights to variants in a group, assuming that rarer variants have larger effects on a disease [5]. This type of tests (often referred to as the Burden test) assume that variants have the same direction of effects on a disease but may significantly lose power when both risk and protective variants are present. The replication-based test accounts for different directions of effects of the variants by grouping variants with the same effects based on their allele frequencies in cases and controls [6]. The C-alpha test also accounts for the mixture of risk and protective variants by testing the distributions of rare variant alleles between cases and controls [7]. The sequence kernel association test (SKAT) uses a regression framework and a variance-component test to consider common and rare variants and different directions of effects [8]. SKAT was further extended to SKAT-O, which finds an optimal weight to combine the Burden test statistic and the SKAT statistic, such that the power for association test can be maximized [9]. SKAT was also extended to several combined tests that assign optimal weights to combine the common and rare variant statistics [10].

Several family-based association tests have been developed recently for rare variants. Most of them were extended from methods developed for case-control design. For example, the weighted-sum approach was applied to the FBAT multimarker test [11]. The Burden and SKAT tests have also been extended to account for relatedness among family members [12], [13]. These tests focused on identifying a group of rare variants associated with a disease and generally assigned higher weight to rare variants and much lower weight to common variants. However, as common and rare variants may both be causal variants [14], common variants with modest effects in a region may not be identified by these methods.

In this work, we propose to use the ordered subset algorithm [15], [16] to select common variants and the variable threshold algorithm [17] to select rare variants. The ordered subset algorithm was originally proposed to identify a subset of families that have the strongest linkage or association signal based on a trait-related covariate. Here we propose a modified algorithm to identify a subset of common variants that have the strongest association signal based on genotypic relative risks of common variants. Based on evolutionary theory, the variable threshold algorithm aims to identify an allele frequency threshold such that rare variants with allele frequencies below the threshold are more likely to be causal [17]. Incorporating both of these algorithms, we use the Pedigree Disequilibrium Test (PDT) [18] to calculate the association test statistics. The statistics for the rare and common variants are combined to form the final test statistic. A simulation study considering several scenarios was conducted to examine the type I error rates for the test. Power studies were performed to evaluate the performance of the test with other existing tests under various simulation scenarios.

## Material and Methods

### The PDT Statistic

We first introduce the PDT statistic as it is used as the fundamental test statistic in the proposed test. An informative nuclear family for the PDT is a family that has at least one affected child and two genotyped parents, where at least one parent is heterozygous. A discordant sibship is informative for the PDT if there is at least one affected and one unaffected sibling with different genotypes. Here we consider families that contain an informative nuclear family and/or an informative sibship. Assume that allele 1 is the minor allele and allele 2 is the other allele at a variant. For a triad (two parents and one affected offspring) in a nuclear family, *T* =  (count of allele 1 transmitted) – (count of allele 1 not transmitted). For a discordant sib pair, *S* =  (count of allele 1 in affected sib) – (count of allele 1 in unaffected sib). Then the PDT statistic *D_i_* for a family *i* is defined as 
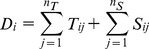
(1)where 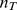 is the number of triads and 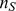 is the number of discordant sib pairs in the family *i*. Assume that we have *N* nuclear families. The single-variant PDT statistic *X* is 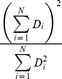, which follows a chi-square distribution with 1 degree of freedom [18]. The null hypothesis is no linkage or no association. Because it considers squared values, the statistic is the same if the minor allele shows a risk or protective effect.

### Estimating Genotypic Relative Risks Using Parental Data

We use genotypic relative risks (GRRs) to select common variants in the proposed test. We modified the approach in [19] to estimating GRRs in family data. Let 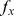 be the penetrance function for the genotype *x* at a variant, where *x* can have values of 0, 1, or 2 based on the minor allele count at the variant. Then the GRRs 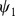 and 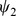 are defined as 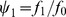 and 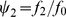, respectively. Assuming Hardy-Weinberg Equilibrium (HWE) and that each family was ascertained with at least one affected sib, four ratios of parental mating types as functions of the GRRs can be defined as [19]:
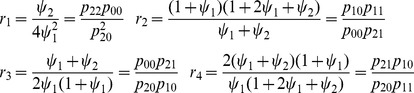
(2)where 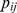 is the probability of a mating type of parents (one has genotype *x* = *i* and the other has genotype *x* = *j*) conditional on the fact that their child is affected. The probability 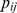 can be estimated using the sample, and estimates of the four ratios (i.e. 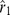, 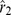, 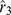, and 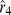) can be obtained. Moreover, if we assume an additive model (i.e., 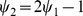 and 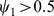), 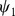 and 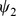 can be estimated based on each of the ratios. Murphy et al. calculated conditional power based on the estimated GRRs and used the conditional power to prioritize SNPs. Their simulation studies suggested that 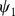 and 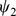 estimated based on 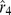 resulted in the highest power for the prioritizing strategy [19]. However, 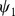 and 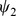 may not have a unique solution or no solution may exist, as the calculations involve solving the square root. In addition, information can be lost if 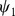 and 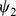 are estimated using only 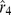. Instead of estimating 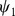 and 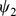 based on only one ratio, we heuristically search for 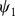 in the range of (0.5, 20), assuming an additive model. Note that 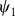 can be less than 1 if the allele has protective effect on the disease. The best estimates of 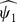 and 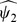 are those whose ratios have the minimum sum of the Euclidean distances to 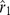, 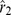, 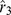, and 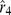. The estimates are unique and always solvable. We use a similar bootstrap approach as in [20] to estimate the variance of the log of 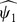. The log of 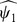 can then be normalized as:



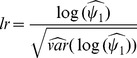
(3)When minor allele frequency is low for a variant, some parental mating types may not be observed in the sample. Then some ratios in equations (2) cannot be estimated. In this case, we only estimate 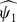 and 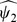 based on the ratios that can be estimated. If all of the ratios cannot be estimated, 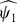 and 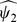 cannot be estimated and *lr* in equation (3) is set as 0. Using simulation studies, we found that 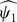 and 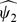 for variants with MAF>0.1 generally can be estimated. For variants with MAF<0.05, 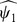 and 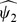 are generally not estimable.

### The Ordered Subset – Variable Threshold – PDT (OVPDT) algorithm

We assume that there are *k* trio families (two parents and one affected offspring). Although we focus our discussions on trios, the method can be generalized to general nuclear family structures. In a sequencing region, assume that there are *n* common variants with minor allele frequency (MAF)>0.05 and *m_t_* rare variants with MAF<*t* for a given allele frequency threshold *t*. The MAF for each variant is estimated from the parental genotypes in the sample. The region can be defined as any set of variants (e.g. variants in exons, introns, gene boundaries, or pathways). The PDT statistic *X* is calculated for each of the common and rare variants.

We use the ordered subset algorithm to select a set of common variants from the *n* variants. The log of relative risk (*lr*) with respect to minor allele is calculated for each of the *n* variants. The absolute values of *lr* are ordered from large to small, and the PDT statistics are ordered based on the rank of their corresponding *lr* values. We use the absolute values of *lr* for ordering so that variants with protective effects can also be in the top rank. Assume that the ordered PDT statistics are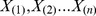. The statistics are added one by one into a subset, and each time a statistic is added into a subset, we calculate a p-value for the sum of statistics in the subset. After all *n* variants are added to the subset, the minimum p-value is calculated as follows:
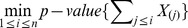
(4)where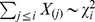, assuming that the *i* variants are independent. The approach in (4) is similar to the adaptive rank truncated product (ARTP) method [21], except that ARTP sorts the SNPs based on their p-values, while our method sorts the SNPs based on their relative risks estimated only based on parental genotypes. As variants may not be independent due to linkage disequilibrium (LD), we will show in our simulations that the proposed test is robust to the assumption of independence. We define the common variant statistic *C* as the statistic corresponding to the minimum p-value if the minimum p-value is less than or equal to 0.05. *C* is defined as 0 if the minimum p-value is larger than 0.05.

We found that the ordered subset algorithm incorporating GRRs is only suitable for selecting common variants. Calculating the ratios in Equations (2) requires parents with homozygous minor alleles, which are often not observed in the sample for rare variants. Therefore, a different strategy is used to select rare variants. We use the variable threshold algorithm to select several sets of rare variants based on different *t*. For a set of *m_t_* variants with MAF<*t*, we calculate the rare variant statistic as follows:


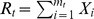
, where *X_i_* is the single-variant PDT statistic for variant *i*. The combined statistic for rare and common variants at the threshold *t* is defined as follows:
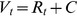
(5)


The OVPDT algorithm is summarized as follows:

1. Calculate the normalized *lr* values for variants with MAF>0.05.
2. Calculate the single-variant PDT statistics for all variants.
3. Calculate the combined statistics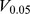, 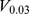, 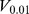, and 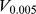 across four thresholds.
4. Permute the transmitted/nontransmitted alleles from parents to siblings simultaneously within each family. Repeat steps 2 and 4 for *K* times. For a permutation *i*, we obtain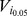, 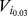, 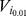, and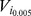.
5. Standardize 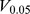, 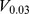, 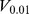, and 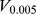 as 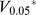, 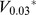, 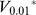, and 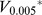 and the permuted statistics as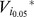, 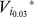, 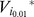, and 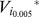 based on the permuted statistics.
6. Define the OVPDT statistic 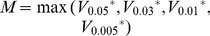, and 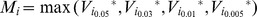 for permutation *i*.
7. The p-value is calculated as ((# of *M_i_*>*M*)+1)/(*K*+1).

We compute the rare variants statistic (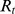) for variants with MAF below each of the four MAF thresholds (i.e., 0.05, 0.03, 0.01, 0.005), and select the maximum statistic from the standardized statistics combining rare and common variant statistics (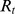
+*C*) across different *t*. Therefore, for rare variants, only variants with MAF below a certain threshold contribute to the statistic *M*. This is based on the observations in the simulation studies in [17] that rare variants below a certain MAF threshold are more likely to be functional than rare variants with MAF above the threshold.

Similar to the permutation strategy used in [22], we randomly permute the transmitted/non-transmitted alleles from parents to siblings in step 4. The transmitted/non-transmitted alleles from parents to all siblings are permuted simultaneously so that the identify-by-descent (IBD) status for alleles between siblings does not change. Therefore, linkage is maintained in the permutations. The permutation of the transmitted/non-transmitted alleles for a family results in a sign change in the PDT statistic for the family. We simultaneously permute the signs of the statistics for variants on the same chromosome to preserve the linkage disequilibrium (LD) structures among the variants. An adaptive permutation strategy [23] is used so that small p-values can be calculated efficiently. Based on the recommendations in Che et al. [23], the permutation procedure is stopped when the number of 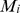 greater than *M* equals 36 and when *K* is greater than 2,000. This strategy guarantees that the standard error in estimating p-values at 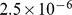 is less than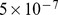.

Because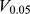, 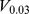, 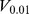, and 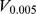 have different distributions, they are not directly comparable in step 6. For a specific 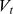, we use its permuted statistics to calculate the mean and standard deviation for the null distribution. Then 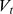 and its permuted statistics are standardized based on the mean and standard deviation in step 5, similar to the procedure used in [24].

### Simulations

We used simulations to evaluate the type I error rates and power for the OVPDT statistic. We used the sequence simulator cosi [25] based on a coalescent model. The best-fitting model provided in the software, which includes parameters such as ancestral population sizes, duration of expansion, migration rates, and mutation rates, was used to generate sequences with an allele frequency spectrum similar to the European and African American populations. Two different sizes of regions, 10 kb and 25 kb, were simulated for each simulation replicate. The 10 kb and 25 kb regions had an average of 198 and 512 variants, respectively.

We used the simulation software SeqSIMLA [26] to generate family data and disease status based on the sequences generated by cosi. The prevalence model in SeqSIMLA, which is based on a logistic penetrance function with odds ratios and prevalence specified by the user, was used in the simulations. The disease prevalence was assumed to be 5%. We estimated allele frequencies based on the sequences from cosi and defined variants with MAF<0.01 as rare variants and other variants as common variants for the simulations. The average numbers of rare and common variants were 166 and 32 in the 10 kb region, while the average numbers of rare and common variants were 429 and 83 in the 25 kb region. The odds ratio for a rare variant with risk effect was a function of its MAF, 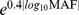, which is the same function used in [12]. As odds ratios for common variants identified by GWAS do not have strong correlations with allele frequencies [27], we randomly generated an odds ratio between 1.05 and 1.3 for a common variant with risk effects, regardless of its allele frequency. A variant with a protective effect had an inverse of the odds ratio generated based on the above methods. Table 1 summarizes the scenarios we used for the type I error and power simulations. For type I error simulations, we considered different sequencing regions (10 kb or 25 kb), sample sizes (500 or 1,000 families), family structures (trios, families with two affected sibs or families with three siblings, with one sib is affected), and the presence of population stratification (Caucasian and African American). For power simulations, the parameters we considered included different sequencing regions (10 kb or 25 kb), proportions of causal variants (10% or 30%), proportions of common and rare variants in the causal variants (100% rare variants, 100% common variants, or 50% common and 50% rare variants), and proportions of risk and protective variants in the causal variants (100% risk variants, or 30% protective and 70% risk variants). One thousand Caucasian trios were simulated for each scenario for the power studies. A total of 20,000 and 1,000 replicates were simulated to calculate the type I error rate and power, respectively, for each scenario.

**Table 1:** Scenarios for type I error and power simulations.

Scenario	Setting
Type I error	
Scen1	10 kb^1^, 500 A^2^, Caucasian^3^
Scen2	10 kb, 1000 A, Caucasian
Scen3	10 kb, 1000 AUU, Caucasian
Scen4	10 kb, 1000 AA, Caucasian
Scen5	25 kb, 500 A, Caucasian
Scen6	25 kb, 1000 A, Caucasian
Scen7	25 kb, 1000 AUU, Caucasian
Scen8	25 kb, 1000 AA, Caucasian
Scen9	10 kb, 700 Caucasian trios and 300 African American trios
Scen10	25 kb, 700 Caucasian trios and 300 African American trios
Power	
Scen11	10 kb, 1000 A, 30% of rare variants are risk variants
Scen12	Same as Scen11. But 30% of the causal variants are changed to protective variants.
Scen13	10 kb, 1000 A, 30% of common variants are risk variants
Scen14	10 kb, 1000 A, 10% of common and 10% of rare variants are risk variants
Scen15	10 kb, 1000 A, 30% of common and 30% of rare variants are risk variants
Scen16	Same as Scen15. But 30% of the causal variants are changed to protective variants
Scen17	25 kb, 1000 A, 30% of rare variants are risk variants
Scen18	Same as Scen17. But 30% of the causal variants are changed to protective variants.
Scen19	25 kb, 1000 A, 30% of common variants are risk variants
Scen20	25 kb, 1000 A, 10% of common and 10% of rare variants are risk variants
Scen21	25 kb, 1000 A, 30% of common and 30% of rare variants are risk variants
Scen22	Same as Scen21. But 30% of the causal variants are changed to protective variants

1 Size of the sequence region.

2 A: two parents and one affected sib; AUU: two parents, one affected and two unaffected sibs, AA: two parents and two affected sibs.

3 Simulated population.

We compared the power of OVPDT with the family-based Burden (FB-Burden) and SKAT (FB-SKAT) tests [12]. FB-Burden uses the weighted-sum approach, which is similar to the FBAT test for rare variants [11]. FB-SKAT extends the SKAT approach to family data. FB-Burden and FB-SKAT use a beta distribution to assign more weight to rarer variants. We also changed the weight function in FB-SKAT so that common variant can receive a larger weight. A default weight for rare variant was used based on the beta distribution, but a flat weight was assigned to common variants equal to the weight for variant with MAF = 0.05, 0.03, 0.01 and 0.005. The FB-SKAT incorporating different flat weights for common variant are referred to as FB-SKAT_0.05, FB-SKAT_0.03, FB-SKAT_0.01, and FB-SKAT_0.005. As the software implementation of FB-Burden and FB-SKAT assumes trios, we only compared the power among different methods using trios.

## Results

**Figure 1:**
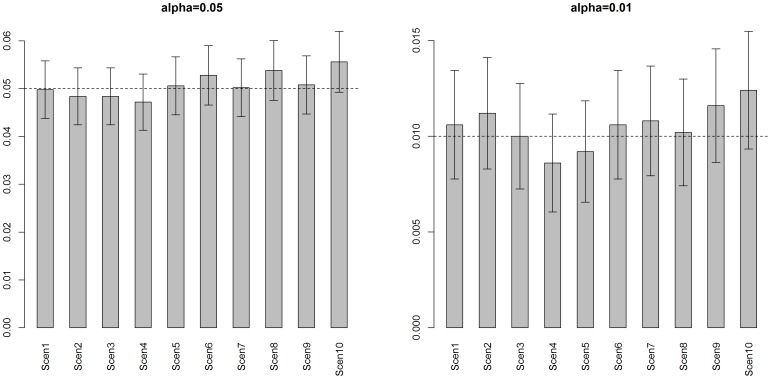
Type I error rates for OVPDT at the 0.05 and 0.01 significance levels. The error bars represent the 95% confidence intervals for the type I error rates.

We show the type I error rates for OVPDT under the 10 scenarios in Figure 1 at the 0.05 and 0.01 nominal levels. OVPDT controls the type I error rates properly at both levels under all scenarios. The 95% confidence intervals contain the nominal levels under all scenarios. Although the genotypic relative risks for common variants were estimated under the assumption that families were ascertained with one affected sib and genotype frequencies were under HWE, OVPDT maintains proper type I error rates for the scenarios in which families had two affected sibs (i.e., Scen4 and Scen8) and for the scenarios in which HWE was violated (i.e., Scen9 and Scen10). Therefore, OVPDT is robust to the violation of the assumptions based on the simulation models. To evaluate the validity of the OVPDT test at more extreme tails, we show the quartile-quartile (QQ) plot for the 20,000 replicates for each scenario in Figure S1. The 20,000 replicates resemble 20,000 genes in real data analysis. As seen in the Figure, the distribution of p-values agrees well with the expected values in each scenario, and the p-values generally fall within the 95% confidence intervals.

The power comparison for OVPDT with FB-Burden and FB-SKAT is shown in Table 2. For Scen11 and Scen17, in which the causal variants are rare and assumed to be risk variants, FB-Burden had more power than OVPDT, followed by FB-SKAT. However, when 30% of the rare causal variants are changed to be protective variants (Scen12 and Scen18), OVPDT had more power than FB-SKAT, while FB-Burden had the lowest power. This is as expected because OVPDT and FB-SKAT take the directions of effects for causal variants into account. For Scen13 and Scen19 where only common variants are responsible for the disease, OVPDT can have much higher power than FB-Burden and FB-SKAT. This is also as expected because lower weights are given to common variants in FB-Burden and FB-SKAT. For all scenarios where the causal variants are a mixture of rare and common variants, OVPDT showed more power than FB-Burden and FB-SKAT. The results demonstrated that considering rare and common variants using different types of algorithms in OVPDT can increase the power of family-based association analysis for sequence data, particularly when the causal variants are mixed with rare and common variants.

**Table 2:** Power comparison of OVPDT with FB-Burden and FB-SKAT at the 0.05 significance level.

Scenario	FB-Burden	FB-SKAT	FB-SKAT_0.05	OVPDT
Scen11	0.815	0.659	0.216	0.716
Scen12	0.470	0.564	0.175	0.608
Scen13	0.179	0.294	0.603	0.436
Scen14	0.364	0.421	0.513	0.519
Scen15	0.750	0.726	0.659	0.757
Scen16	0.373	0.703	0.722	0.801
Scen17	0.918	0.826	0.344	0.854
Scen18	0.596	0.740	0.226	0.776
Scen19	0.240	0.448	0.722	0.611
Scen20	0.535	0.613	0.678	0.722
Scen21	0.658	0.811	0.759	0.853
Scen22	0.337	0.871	0.829	0.896

We also show the power for FB-SKAT_0.05 in Table 2 for the FB-SKAT test incorporating different weight functions for rare and common variants. The power for FB-SKAT_0.03, FB-SKAT_0.01, and FB-SKAT_0.005 is not shown because we found that FB-SKAT_0.05 always had more power than these tests in our simulation scenarios. When the causal variants are all rare (i.e. Scen11, Scen12, Scen17, and Scen18), FB-SKAT_0.05 had significantly lower power than OVPDT, FB-Burden and FB-SKAT. FB-SKAT_0.05 can have the highest power when the causal variants are all common (i.e. Scen13 and Scen19). This is not surprising as a higher weight is given to common variants in FB-SKAT_0.05. When the causal variants are mixed with common and rare variants, the power for FB-SKAT_0.05 and FB-SKAT is similar, but lower than the OVPDT. In general, the OVPDT still has the highest power in most of the scenarios.

**Figure 2:**
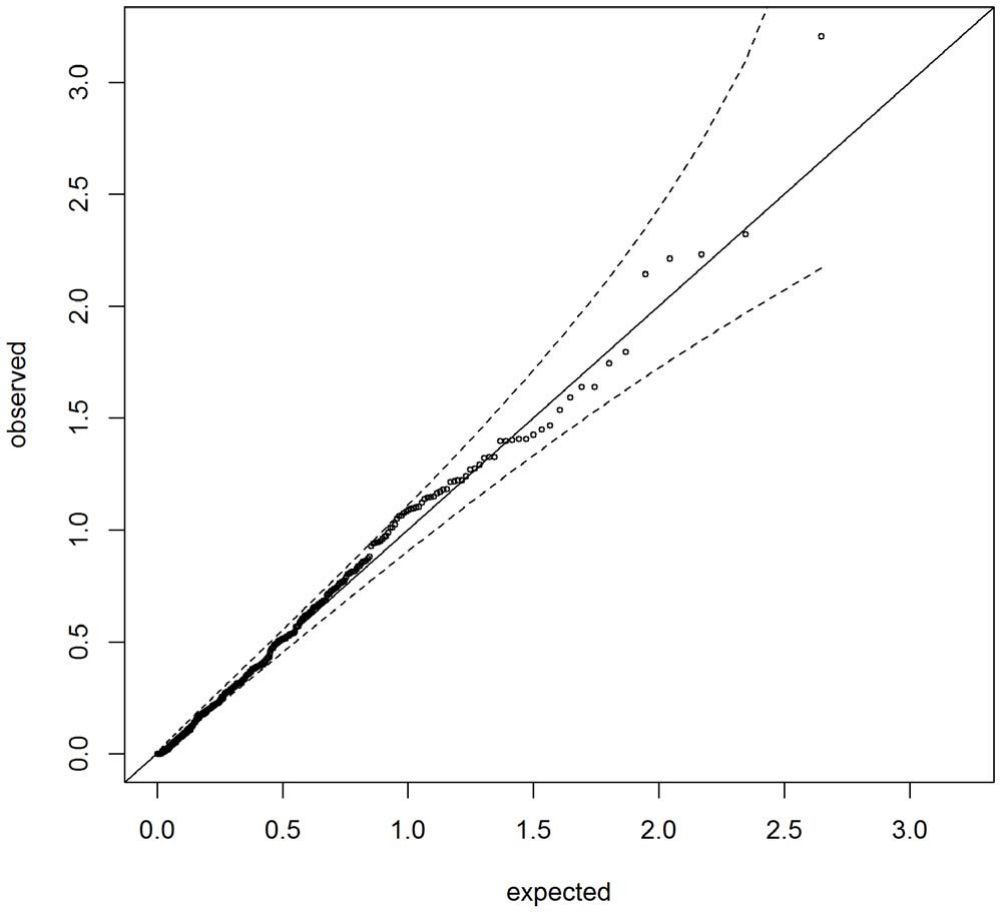
QQ plot for the pathway-based analysis using the GOAL study data.

We applied OVPDT to the Genomic Origins and Admixture in Latinos (GOAL) study dataset. The dataset consists of 25 trios genotyped on Illumina Human Exome Beadchips. Variants with missing rates>10% were removed. Variants with HWE test p-values <0.001 were also removed. The overall genotyping rate was 99.97% and there were 55,569 polymorphic variants in the data. The 25 trios do not have disease status as the aim of the GOAL project is to investigate the haplotype structures in Hispanics. Therefore, we assumed the child was affected (but affection status was unrelated to genotypes) and parents were unaffected in each trio. Although the affection status was artificially determined, the analysis results can be used to evaluate the validity of the test in real data. Because most of the genes contain only one or a few variants, we performed pathway-based analysis. We downloaded the entire gene sets from the Molecular Signatures Database (MSigDB) from the GSEA website (http://www.broadinstitute.org/gsea). We extracted the gene sets that each pair of the gene sets do not overlap for more than 5% of the genes. A total of 442 gene sets were tested. The QQ plot for the analysis results is shown in Figure 2. The p-values fall within the 95% confidence intervals in the plot, which demonstrates that the OVPDT maintains a valid test in real data.

## Discussion

We have developed a novel family-based association test, OVPDT, which considers both common and rare variants and the directions of effects of the variants. We performed a simulation study to evaluate the properties of the proposed tests. Our simulation results showed that the proposed test has correct type I error rates under all simulation scenarios. We also used simulations to compare the power of the proposed test with FB-Burden and FB-SKAT. The simulation results showed that, when the causal variants are a mixture of rare and common variants, OVPDT is more powerful than FB-Burden and FB-SKAT.

OVPDT takes advantage of the unique property of family data that parents can be used to calculate the relative risks for common variants. The calculations of the relative risks are based on the assumptions that families were ascertained with at least one affected sib and the genotype frequencies were under HWE. Using simulations, we demonstrated that type I error for OVPDT was maintained at the expected levels even when the two assumptions were violated. The calculations of the relative risks can be extended to more general family structures, such as families with more than one affected sib or families with missing parents. Different ratios of parental mating types which are independent from population allele frequencies need to be derived, which can be accomplished by sophisticated mathematical packages [19].

The relative risks are used in both the two-stage method in Murphy et al. [19] and our method to rank the variants. In the first stage of the two-stage method by Murphy et al., the genotypic relative risks are used to calculate the conditional power of the FBAT test [28] for each variant. In the second stage, variants are ranked by their conditional power and a weighted Bonferroni approach is applied to the ranked p-values to determine whether a variant is significant. In our method, the genotypic relative risks are normalized by their variance and are incorporated in the ordered subset algorithm to select a promising subset of variants. Therefore, the general purpose of the two-stage method and our method is to use the genotypic relative risks to prioritize the variants. However, two different algorithms (i.e. the weighted Bonferroni approach and the ordered subset algorithm) are used after the variants are ranked by their relative risks, because the goal of the two-stage method is to identify association for single variants, while our goal is to identify a subset of promising common variants.

The common and rare variant statistics contribute equally to the OVPDT statistic. Different weights can be assigned to the common and rare variant statistics to increase the power for the test, when the overall effect sizes for the common and rare variants are very different [10]. Similar adaptive approach to determining the optimal weights as used in [10] can potentially be applied to the OVPDT algorithm. However, the adaptive approach relies on the fact that the distributions for the common and rare variants are known, which is not the case for OVPDT. More research will be needed to decide the optimal weights for the common and rare variant statistics in OVPDT.

We used a permutation procedure to approximate the distribution for the proposed test. As permuting transmitted and non-transmitted alleles in the permutation procedure does not change parental mating types, the relative risks and their variance, which are calculated based only on parental mating types, do not need to be recalculated in the permutation. We do not specifically model LD in the test statistic by taking the sum of the individual statistics. However, LD structures among variants were properly maintained in the permutation. Therefore, although LD is not specifically modeled, the presence of LD does not affect the validity of the test.

The permutation procedure has a nice property in that permuting the transmitted and non-transmitted alleles from parents to siblings simply results in a sign change for the PDT statistic for each family. However, this property only holds for nuclear families. For extended pedigrees, permuting the transmission and non-transmission alleles is challenging, especially when there are missing parents. Linkage also needs to be considered when there are multiple affected siblings [29] in the permutation. Alternatively, a Monte-Carlo simulation method, as used in MERLIN [30], can be used to generate null data. This is our future work to extend the proposed methods to extended pedigrees based on a Monte-Carlo simulation procedure.

In conclusion, OVPDT will be useful in detecting both common and rare variant effects on a complex disease based on family sequencing data. The proposed test provides an alternative test to the currently available family-based rare variant tests. We have implemented the proposed test in a software package, OVPDT, with C++. POSIX Threads (Pthreads) in C++ are used to parallelize the code. The program can finish the analyses based on 2,000 permutations for the simulated 10 kb and 25 kb regions in 1,000 nuclear families in 8.6 and 17.3 seconds, respectively, on a Linux server with Xeon 2.0 GHz CPUs with 8 threads. Therefore, the program can perform genome-wide gene-based analysis in a reasonable time frame. The program can be downloaded for free at http://ovpdt.sourceforge.net.

## Supporting Information

Figure S1 **QQ plots for the type I error simulations.**
(TIFF)
