# Supplementary figures and images for: Family-Based Association Test Using Both Common and Rare Variants and Accounting for Directions of Effects for Sequencing Data
Source: PLoS One. 2014 Sep 22;9(9):e107800. doi: 10.1371/journal.pone.0107800 (PMC4171487; doi:10.1371/journal.pone.0107800)

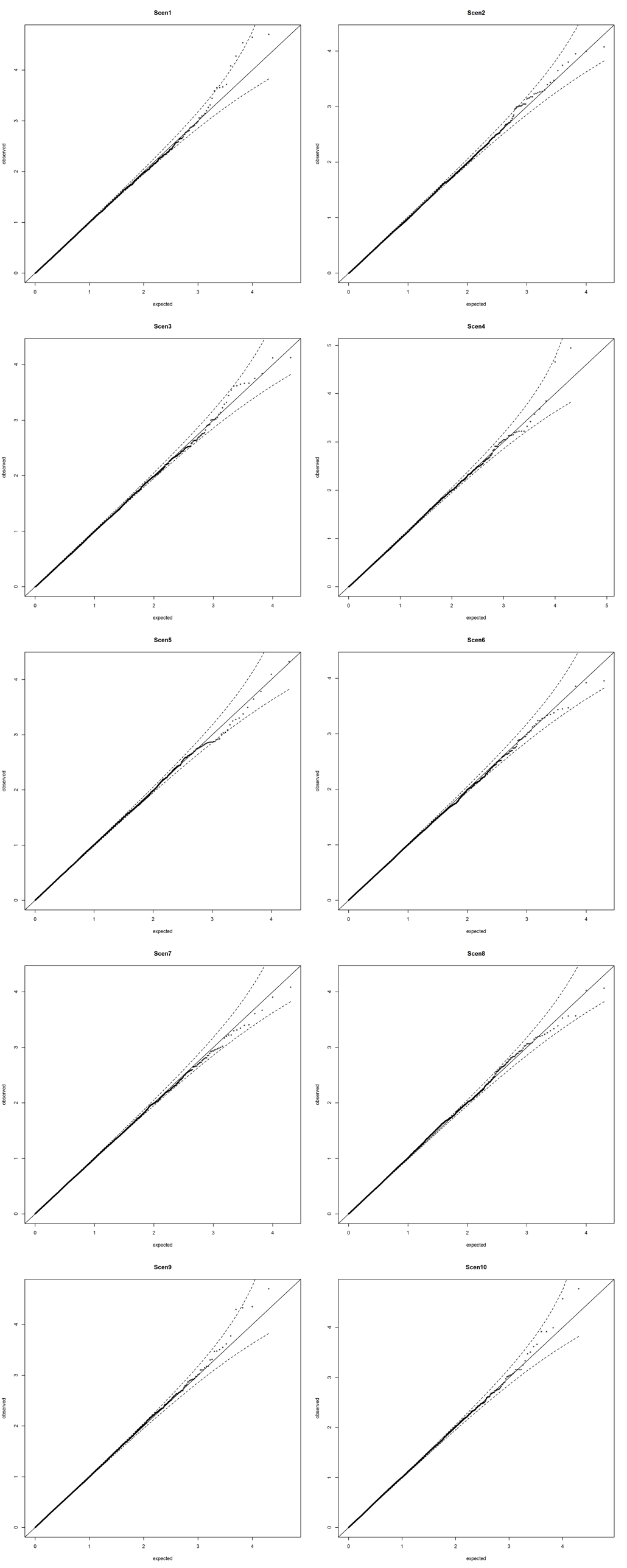

Supplement: Figure S1 — QQ plots for the type I error simulations. (TIFF) [file pone.0107800.s001.tiff]
